# Predictors of mortality caused by hypothermia in Japan: Author's reply

**DOI:** 10.1002/jgf2.441

**Published:** 2021-05-05

**Authors:** Yasuharu Tokuda, Naoto Ishimaru

**Affiliations:** ^1^ Muribushi Project for Teaching Hospitals Okinawa Japan; ^2^ Department of General Internal Medicine Akashi Medical Center Hyogo Japan

To the Editor,

We thank the correspondents for critically appraising our retrospective study of predictors of mortality caused by hypothermia in Japan.^1^ Their concerns regarding the methodology for estimating prognostic factors of hypothermia in Japan are indeed valid.

As mentioned, the study included patients who had cardiac arrest and those who died in the emergency room (ER), and this could be reverse causality. We needed to use the same inclusion criteria as the study by Elbaz et al because our study aimed to validate the in‐hospital mortality prediction score developed in their original study.^2^ Actually, none of our patients died in the emergency room, and the one patient with cardiac arrest on arrival recovered from cardiac arrest and shock and survived for 2 days.

It was also suggested that the results of the analysis after adjusting for optimal cutoff points should have been included in the study. The C‐statistic of the in‐hospital mortality prediction score upon adjusting the cutoff values was 0.80 (95% confidence interval, 0.67‐0.92). Just for reference, the C‐statistic in the initial analysis was 0.70 (0.55‐0.84), which rose to 0.81 (0.69‐0.92) after adjusting the cutoff values and selection of the variables.^3^

The final comment was on the selection of the variables, suggesting that logistic regression analysis using multiple explanatory variables may be inappropriate for the small sample size in our study. There is a concern about overfitting when it comes to the number of events per variable less than 10, as in our study.^4^ To avoid overfitting, as we stated in the statistical analyses section in our report,^3^ we adopted a backward algorithm with Akaike's information criteria (AIC) in stepwise regression.^5^ The AIC was 75.03 upon logistic regression analysis, 69.00 after adjusting the cutoff values, and 63.63 after adjusting the cutoff values and selection of the variables.

## CONFLICT OF INTEREST

The authors have stated explicitly that there are no conflicts of interest in connection with this article.
